# Daylight exposure and circadian clocks in broilers: part I—photoperiod effect on broiler behavior, skeletal health, and fear response

**DOI:** 10.1016/j.psj.2023.103162

**Published:** 2023-10-02

**Authors:** Sha Jiang, Yuechi Fu, Heng-wei Cheng

**Affiliations:** ⁎ Joint International Research Laboratory of Animal Health and Animal Food Safety, College of Veterinary Medicine, Southwest University, Chongqing 400715, China; † Department of Animal Sciences, Purdue University, West Lafayette, IN 47907, USA; ‡ USDA-Agricultural Research Service, Livestock Behavior Research Unit, West Lafayette, IN 47907, USA

**Keywords:** chicken, photoperiod, behavior, skeletal health, fear

## Abstract

The aim of this study was to examine effects of various daylight exposure during the 24-h light-dark (**L-D**) cycle on growth performance, skeletal health, and welfare state in broilers. Environmental photoperiod and related circadian clock, the 24-h L-D cycle, are important factors in maintaining productive performance, pathophysiological homeostasis, and psychological reaction in humans and animals. Currently, various lighting programs as management tools for providing a satisfactory environmental condition have been used in commercial broiler production. Four hundred thirty-two 1-day-old Rose 308 broiler chicks were assigned to 24 pens (18 birds/pen). The pens were randomly assigned to 1 of 4 thermal and lighting control rooms, then the birds were exposed to (*n* = 6): 1) 12L, 2) 16L, 3) 18L, or 4) 20L at 15 d of age. Lighting program effects on bird body weight, behavioral patterns, bone health, and stress levels were evaluated from d 35 to d 45, respectively. The birds of 12L as well as 16L groups, reared under short photoperiods close to the natural 24-h L-D cycle, had improved production performance, leg bone health, and suppressed stress reaction compared to the birds of both 18L and 20L groups. Especially, 12L birds had heavier final body weight and averaged daily weight gain (*P* < 0.05), higher BMD and BMC with longer and wider femur (*P* < 0.05), lower H/L ratio (*P* < 0.05), and more birds reached the observer during the touch test (*P* < 0.05) but spent shorter latency during the tonic immobility test (*P* < 0.05). Taken together, the data suggest that supplying 12 h as well as 16L of daily light improves performance and health while decreasing stress levels in broilers, making it a potentially suitable approach for broiler production.

## INTRODUCTION

Environmental photoperiod, such as the 24-h L-D cycle, is an important factor providing information of daily sunlight change (daytime and nighttime) for maintaining and synchronizing the physiological and behavioral homeostasis in humans and animals, such as daily rhythms-associated food intake, nutrient metabolism, body temperature, productive performance, physiological function, immune regulation, and mood reaction as well as sleep-wake cycle (Nicholls et al., 2019; Gordon et al., 2020; Zeng et al., 2022). Light pollution, such as aberrant light at night caused desynchrony (circadian misalignment), has become a potential risk factor for the health and welfare of humans and animals (Fishbein et al., 2021; Burt et al., 2023; Wang et al., 2023). The long-day (photoperiod) exposure causes sleep-wake disturbance (Aulsebrook et al., 2018). In humans, sleep disturbance is an important contributor to various adverse health and welfare consequences, such as gastrointestinal diseases, metabolic disorders, and mental distress (Bechtold et al., 2010; Paksarian et al., 2020; Khan and Aouad, 2022). The functional regulation of the 24-h light-dark cycle has been recognized as “circadian medicine” or “circadian health” in humans (Kramer et al., 2022). Similarly, sleep disruption may affect lighting, synchronizing their daily rhythms of physiological and behavioral processes, reducing feed intake, body weight gain, and immunity in birds (Alaasam et al., 2021). In nature, wild chickens, as diurnal animals, are under the regulation of daily lighting changes and perform their activities at a particular time in a 24-h light-dark cycle, being active and inactive during the day and night, respectively (Wikelski et al., 2008; Rani and Kumar, 2013). In modern commercial poultry meat farms, broilers as well as layers domesticated from red Junglefowl (*Gallus gallus*) have been removed from being exposed to the natural environments to human-made indoor rearing systems, such as using artificial lighting programs which do not mimic the daily changes of sunlight. In addition, commercial broilers are usually housed in intensive environments, several 10,000 birds in 1 barn, consequently, the birds moving within the restricted environment may disrupt their social and resting behaviors, resulting in social stress and sleep disorders, that is, short sleep, sleep fragmentation, or suboptimal sleep quality. Furthermore, to meet the continuously increased global animal protein demand, broilers have been selected based on feed efficiency and growth rate as well as breast meat yield, reaching an average of 6 pounds of live weight by approximately 6 wk (Kabir and Islam, 2021). To reach and maintain the production goal, several artificial light programs with prolonged photoperiods have been used. Broilers are often reared under continuous or near-continuous dim lighting, transforming the nighttime environment to the daytime environment to stimulate growth, by which it provides more time for feed intake, consequently, increasing production and economic profile (Yang et al., 2015; Wu et al., 2022). However, increased exposure to artificial light at night and eating at inappropriate time of the day may mask natural photoperiodic cues and reach a state of allostatic overload perturbing physiological and behavioral homeostasis through the misalignment of daily rhythms (the natural light-dark cycle) when birds try to adapt to the artificial environments by resetting internal circadian clock at the cellular and tissue levels. These changes lead to internal desynchrony with a chronic local or systemic low-grade inflammation (a long-time persisting condition causing tissue and organ damage), potentially resulting in various diseases, such as ascites syndromes (water belly) and musculoskeletal disorders (leg problems) (Baghbanzadeh and Decuypere, 2008; Schwean-Lardner et al., 2013; Khajali, 2022).

In the past decades, it has been recognized that light management is one of the critical environmental factors affecting poultry production (Olanrewaju et al., 2006; Thaxton et al., 2016; Shynkaruk et al., 2022; Kang et al., 2023). Numerous studies have investigated the photoperiodic effects on broiler health and welfare, and various photoperiodic regimes have been used, for example, continuous light (**L**) at 14L, 17L, 20L, and 23L from d 6 to 31 (Shynkaruk et al., 2019, 2022), 8L, 18L, and 24L from d 7 to 35 (Kim et al., 2022), 8L and 23L from d 8 to 56 (Olanrewaju et al., 2019), and 16L, 22L, and 24L from d 2 to 26 (Sanotra et al., 2002) as well as various intermittent light, such as 4L:2D (Kim et al., 2022), 2L:2D (Olanrewaju et al., 2018), and 1L:3D:1L:3D:1L:3D:1L:3D:2L:6D (Rodrigues and Choct, 2019), and combined continuous light with intermittent light based on bird age, 24L:0D at d 0 to 6; 16L:8D at d 7 to 13; 12L:4D:2L:6D at d 14 to 20; 12L:4D:3L:5D at d 21 to 27, 12L:4D:4L:4D at d 28 to 41, and 13L:3D:5L:3D at d 42 to 45 (Nelson, 2020). Although significant progress has been made in understanding the photoperiodic effects on broiler production, health, and welfare, inconsistent results have been reported, increase, decrease, or not change, which is affected by multiple factors without fully being understood. In addition, the lighting program should be simply implemented. Based on the outcomes, continuous lighting programs in broiler production have been barn and requested to provide at least 8-h darkness by the United Kingdom (DEERA (Department for Environment Food and Rural Afairs), 2018) or 6 h total darkness with at least uninterrupted 4 h by European Union (2007). With a similar goal, it has been recommended to provide 23L at d 0 to 7 and 18L or 20L after d 7 by Aviagen (2018). However, the requirement for bird sleep could be higher than recommendations at certain growing points (Blokuis, 1983; Olanrewaju et al., 2006), and longer daytime (23L vs. 20L, 17L, and 14L) increases the risk of birds suffering from sleep fragmentation and reduces welfare (Schwean-Lardner et al., 2014). Therefore, it has become critical to develop an optimal lighting program for improving broiler health and welfare (Kim et al., 2022). The aim of this study was to investigate the effects of various photoperiods on broiler BW, health, and welfare state as well as the underlying mechanisms with the goal of identifying an optimal program to apply. The immediate aim of part 1 was focused on lighting program effects on broiler BW, skeletal strength, stress response, and behavioral exhibitions.

## MATERIALS AND METHODS

The project was approved by the Animal Care and Use Committee of Purdue University (PACUC Protocol Number:1712001657), and birds were housed at the Purdue poultry farm (West Lafayette, IN) in accordance with the Guide for the Care and Use of the Agricultural Animals in Research and Teaching of the Federation of Animal Science Societies (ASAS, 2020).

### Experimental Design and Animal Management

One-day-old Ross 308 broiler chicks (*n* = 432) were weighed and assigned to 24 pens (18 birds per pen at 110 cm × 110 cm) with equal body weight among the pens. The pens were assigned to 4 thermal and lighting control rooms at the Poultry Unit (Purdue University, West Lafayette, IN). The rooms were then randomly assigned to 1 of 4 photoperiod treatments, starting at 15 d of age (*n* = 6): The birds were exposed to: 1) 12D:12L, to minimize the natural photoperiod of about 12-h light and 12-h darkness cycle (the photoperiod was 0600–1800); 2) 16L:8D (0600–2200); and 3) 18L:6D (0600–0000), to meet the requirements proposed by Aviagen and European Union, respectively; and 4) 20L:4D (0600–0200), to mimic the current management practice used by the most of the commercial broiler industries in the United States. Before d 15, the lighting programs were: 24L:0D at 30 lux at d 1, reduced gradually to 23L:2D at 30 lux from d 2 to 7, adjusted gradually until reaching the final expected photo schedule at 5 to 10 lux at d 14, then maintained until 45 d of age, which was designed based on the published Rose Broiler Management Handbook (Aviagen, 2018) and the recommendations (Donald et al., 2000).

The room temperature was 34°C until d 3, gradually reduced until reached 21°C to 24°C, then maintained until d 45. Food and water were provided ad libitum through the experiment. The general management, including vaccination, dietary formulation, and nutrient contents, was followed the protocol reported previously (Tuell et al., 2020a,b).

### Growth Performance and Sample Collection

At d 35 and d 43, each sampled broiler (*n* = 6 at 12 birds, 2 birds/pen × 6 pens at each time point) was randomly picked, weighed, then sedated by injection of sodium phenobarbital (30 mg/kg BW, iv; Sigma-Aldrich, St Louis, MO) via the brachial vine within 2 min of removed from the home pen. Following euthanasia, a 5-mL blood sample was collected through the brachial vein of each sampled bird using an EDTA-coated tube. The blood samples were centrifuged at 700 × *g* for 15 min at 4°C, then plasma was separated and stored at −80°C until further analysis. The birds were euthanized immediately after bleeding by cervical dislocation. The left tibia and femur were collected and placed in individual plastic bags, then kept at −20°C until assayed (Yan et al., 2020).

### Behavioral Observations

Video cameras were set up inside the rooms and bird behaviors were observed by 2 observers using the 5-min scan sampling method (Altmann, 1974; Mack et al., 2013; Wang et al., 2018b). The major behavioral ethogram of broilers was developed based on the ones published previously (Table 1) (Baxter et al., 2018; Wang et al., 2018b; Meyer et al., 2020). The birds performing posture-related behaviors (dustbathing, locomoting, sitting, standing, and sleeping) at 12:00 to 13:00, and eating and drinking were recorded 1 h after lighting on (6:30–7:30) and 1 h before lighting off based on lighting programs, when birds show the most activity based on previous 24-h observations (Cheng and Jefferson, 2008). The behavioral observations were conducted during 2 growing phases, wk 3 to 4 (grower phase) and wk 5 to 6 (finisher phase). Scan sampled postures were calculated with the following formula: the number of birds spent in each behavior/the total bird number during the observation time × 100%. All occurrence sampled foraging behaviors were calculated with the following formula: the time spent in one behavior/the total time spent in all behaviors during the observation time. For each behavior, the data collected daily were averaged for the statistical analysis (Wang et al., 2018b; Weeks et al., 2000; Snyder et al., 2022). The interobserver agreement was 95%.

**Table 1:** Behavioral ethogram^1^.

Behavior	Definition
Drinking	Bird's beak is in contact with drinker
Dustbathing^2^	Bird pecks and scratches at the litter material, then squats down in the substrate, and follows an organized sequence of behavior patterns (combined both preening and scratching behavior)
Eating	Bird's head is located inside feeder
Locomoting^3^^,^^4^	Bird is in the process of taking at least 2 steps including scratching the litter walking, and running
Sitting	Bird sits resting with its abdomen on the floor. Without space is visible between the bird and floor
Standing	Both feet but without other body part contacted with the floor
Sleeping	Bird has eyes closed and the head is tucked into the feathers above the wing base or even behind the wing. It can be performed in a sitting as well as a standing position. While standing, a slight crouching posture is shown with the tail is down

1 Wang, W. C., F. F. Yan, J. Y. Hu, O. A. Amen, and H. W. Cheng. 2018. Supplementation of Bacillus subtilis-based probiotic reduces heat stress-related behaviors and inflammatory response in broiler chickens. J. Anim. Sci. 96:1654–1666.

2 Baxter M., Bailie C. L., and O'Connell N. E. 2018. Evaluation of a dustbathing substrate and straw bales as environmental enrichments in commercial broiler housing. Appl. Anim. Behav. Sci. 200:78–85.

3 Meyer M. M., Johnson A. K., and Bobeck E. A. 2020. Development and validation of broiler welfare assessment methods for research and on-farm audits. J. Appl. Anim. Welf. Sci. 23:433–446.

4 Snyder A. M., S. P. Riley, C. I. Robison, D. M. Karcher, C. L. Wickware, T. A. Johnson, and S. L. Weimer. 2022. Behavior and immune response of conventional and slow-growing broilers to Salmonella Typhimurium. Front. Physiol. 13:890848.

### Skeletal Health

#### Bone Physical and Structural Characteristics

The tibia and femur were measured for bone mineral density (**BMD**), bone mineral content (**BMC**), and bone area using a dual-energy x-ray absorptiometry (Norland Medical Systems Inc., Fort Atkinson, WI) as previously described (*n* = 6, 2 birds per pen, total 12 birds per treatment) (Hester et al., 2013). The BMD was calculated as BMC divided by the area of the bone. After scanning, all the bones were boiled for 5 min, then the soft tissues including meat, connective tissue, and the fibula bone were removed, and the bone length and width were determined using a digital micrometer (Coolant Proof Micrometer Series 293, Mitutoyo America Corp., Aurora, IL) (Yan et al., 2020).

#### Bone Strength

The test sequence of birds was similar to the one used for the fear-related behavioral test, repeating the cycle of 12L:12D, 16L:8D, 18L:6D, and 20L:4D groups until the end of each test, to minimize the potential effects of circadian variations on the examined behaviors. a) Gait Score (**GS**). At d 36, 3 broilers per pen (*n* = 6, 18 birds per treatment) were randomly carefully fenced at a corner of a pen without much disturbance, then each bird was individually released to walk out with scored (Vasdal et al., 2018; Rasmussen et al., 2022). The 3-point GS system (0 = normal gait, 1 = gait with obvious sickness, and 2 = gait with severe sickness) was used as described previously (Webster et al., 2008; Yan et al., 2018; Mohammed et al., 2021b). To avoid using the same birds repeatedly, the birds were marked with different color leg bands after the test. At d 44, the procedure was repeated. b) Latency to Lie (**LTL**). At d 36 and d 44, 3 unmarked birds (*n* = 6, 18 birds per treatment) were randomly used to perform the test (Berg and Sanotra, 2003; Yan et al., 2020). The test was stopped if a broiler still stood after 600 s and the observation of 600 s was recorded.

### Stress and Fear Indicators

#### Heterophil to Lymphocyte Ratio

One hundred leukocytes on each duplicate slide stained within Wright's stain were examined at 2,000 × magnification (total of 200 cells per bird) by using a double-blind design (Cheng et al., 2001). Heterophils and lymphocytes were identified based on their characteristics described by Campbell (1988), from which the heterophil to lymphocyte (**H:L**) ratio was calculated (Gross and Siegel, 1983).

#### Fear-Related Behaviors

To minimize the potential effects of circadian variations on the flowing neurohormone synthesis and on the examined behaviors, the following tests were performed by repeating the cycle of 12L:12D, 16L:8D, 18L:6D, and 20L:4D groups until the end of each test. a) Tonic Immobility (**TI**). A TI test was conducted following the previously published protocol (Zulkifli and Siti Nor Azah, 2004; Dennis et al., 2013; Mohammed et al., 2021a). Briefly, at d 45, 2 unmarked birds were randomly used for the test (*n* = 6, 2 birds per pen, 12 birds per treatment). Each of the tested birds was laid in a cradle upside down and held with slight pressure for 5 s to initiate a state of TI. When pressure was removed, the duration of immobility was measured. If the bird righted itself in less than 10 s, the restraining procedure was repeated. The duration of TI was considered 0 s if TI was not induced after 3 attempts, while the birds were removed from the cradle after 900 s if no attempt to right themselves was made. b) Touch Test. The touch test was carried out by following the published methods (Vasdal et al., 2018; Mohammed et al., 2021b). Briefly, at both d 36 (*n* = 6, 16 birds/pen) and d 44 (*n* = 6, 14 birds/pen), an observer entered the pen and gently sat down facing the birds at 1 of the 2 locations (at the far end and near the entrance), waited for 2 min, then tried to touch the birds that were in reach. Thus, no birds were behind the observer. The mean of touched birds at the 2 locations was calculated per pen for statistical analysis.

### Statistical Analysis

Behavioral, growth performance, and bone strength parameters were analyzed by repeated measures, and bone mineral parameters and stress indicators (TI and H/L ratio) were analyzed by one-way ANOVA of the mixed model procedure of SAS 9.4 software (SAS Institute Inc., Cary, NC) was used for the data analysis with photoperiod program as the fixed effect. The experiment unit was the pen (*n* = 6), and the bird number used for each test served as a subsample. The BW was used as a covariate for the measure of bone mineralization and bone length and width when necessary (Steel et al., 1997). The averaged mean of each parameter collected from the birds was presented for the statistical analysis due to its coefficient variation (**CV**) was less than 15%. The normality of the data was checked using the Shapiro-Wilk test, and the transformation of data was performed when variances were not homogeneous (Steel et al., 1997). Statistical trends were similar for both transformed and untransformed data; therefore, the untransformed least square means and the standard error of the mean (**SEM**) were presented. The Benjamini and Hochberg (1995) method was used to control the false discovery rate due to multiple comparisons and the Tukey-Kramer test was used to partition any significant differences among the least square means due to treatment effects. Statistical significance was set at *P* ≤ 0.05, and the trend was set at 0.05 < *P* ≤ 0.10.

## RESULTS

### Growth Performance

The effects of photoperiod on broiler growth performance are presented in Table 2. Overall, final BW and averaged body weight gain (**A**W**G**) were higher in the birds of the 12L group than those of 20L group (*P* < 0.05). At d 14, before the lighting treatment, there was no difference in the BW (*P* > 0.05), while a tendency of heavier BW at d 20 (5 d after lighting treatment) was found in the 20L group compared to the 12L group (*P* = 0.06). The difference was reversed at d 35 (20 d after the lighting treatment) (*P* < 0.05) and onward to d 43 (28 d after the lighting treatment) which was 3,304 g > 3,246 g > 3,206 g > 3,157 g; 12L > 16L > 18L > 20L (*P* < 0.05). Both 12L and 16L birds had heavier AWG than 20L birds but not 18L:6D birds (*P* < 0.05).

**Table 2:** Effects of photoperiods on body weight and period body gain in broilers during wk 1 to 4 (d 14–43) postlight treatments.

Treatment	D 14	D 20	D 35	D 43	D 15–43
	BW (g)	BW (g)	BW (g)	BW (g)	AWG (g/d)
12L:12D	393.3	769.0^B^	2278.6^a^	3304.4^a^	100.4^a^
16L:8D	388.2	774.4^AB^	2216.6^ab^	3245.6^ab^	98.5^a^
18L:6D	388.1	779.1^AB^	2236.2^ab^	3206.2^b^	97.2^ab^
20L:4D	406.5	784.7^A^	2198.1^b^	3156.8^c^	94.8^b^
SEM	7.2	9.7	26.7	32.6	1.5
*P* value	0.685	0.062	0.022	0.006	0.047

Means within a column with different superscripts are different at:

A,B 0.05 *≤ P* < 0.01.

a–c *P* ≤ 0.05. All means are reported as means ± SEM, *n* = 6 at 12 birds (2 birds/pen × 6 pens). Abbreviations: AWG, average daily weight gain; BW, body weight.

### Behavioral Patterns

There were no lighting effects on bird eating and drinking during wk 3 to 4 (1–2 wk after the lighting treatment. *P* > 0.05, Table 3). However, 12L and 16L birds showed more active (locomoting, i.e., walking and running) as well as standing behavior than the birds of both 18L (*P* < 0.05) and 20L (*P* < 0.05) groups during the observation times. In addition, 12L birds also showed more dustbathing behavior than 20L birds (*P* < 0.05). However, 20L birds but not 18L:6D birds spent more time to sit and to sleep compared to both 16L and 12L birds (*P* < 0.05). During wk 5 to 6 (3–4 wk after the lighting treatment), 12L birds spent more time to eat (*P* < 0.05) and drink (*P* = 0.06) than 20L birds. In addition, both 12L and 16L birds spent more time to stand than 20L birds (*P* < 0.05); while 20L birds spent more time to sit compared to both 12L and 16L birds (*P* < 0.05). In addition, 20L and 18L birds spent more time to sleep than both 16L (*P* < 0.05) and 12L birds (*P* < 0.05).

**Table 3:** Effects of lighting programs on behavioral patterns of broilers.

Behaviors	Wk 3–4					Wk 5–6				
(%)	12L:12D	16L:8D	18L:6D	20L:4D	*P* value	12L:12D	16L:8D	18L:6D	20L:4D	*P* value
Eating	24.6 ± 0.5	26.4 ± 0.6	24.0 ± 0.8	23.0 ± 1.1	0.92	26.5 ± 0.6^a^	23.7 ± 0.8^ab^	22.8 ± 0.7^ab^	20.5 ± 0.8^b^	0.03
Drinking	7.7 ± 1.2	9.9 ± 1.3	6.1 ± 0.8	5.2 ± 0.9	0.71	7.9 ± 1.1^A^	6.6 ± 0.8^AB^	5.2 ± 0.7^AB^	4.6 ± 0.8^b^	0.06
Locomoting	35.5 ± 2.8^a^	29.5 ± 3.6^a^	21.7 ± 2.7^b^	18.9 ± 2.2^c^	0.001	19.2 ± 2.5	18.5 ± 2.1	15.4 ± 1.3	13.8 ± 1.8	0.72
Dustbathing	6.2 ± 0.3^a^	4.2 ± 0.2^ab^	1.8 ± 0.2^ab^	1.3 ± 0.2^b^	0.04	3.6 ± 0.8	2.7 ± 0.3	1.3 ± 0.6	0.8 ± 0.6	0.82
Sitting	6.0 ± 0.97^b^	8.3 ± 1.02^b^	15.6 ± 1.4^ab^	17.6 ± 2.1^a^	0.02	16.2 ± 1.2^b^	18.7 ± 1.2^b^	22.3 ± 1.3^ab^	23.6 ± 1.5^a^	0.04
Standing	29.5 ± 1.1^a^	26.9 ± 1.1^a^	20.0 ± 1.3^b^	18.1 ± 1.1^b^	0.03	18.4 ± 1.2^a^	18.2 ± 1.2^a^	13.5 ± 1.2^ab^	12.5 ± 1.2^b^	0.05
Sleeping	4.9 ± 0.3^b^	5.8 ± 0.3^b^	8.8 ± 0.5^ab^	10.1 ± 1.1^a^	0.02	6.7 ± 0.5^c^	9.1 ± 1.1^b^	14.7 ± 1.3^a^	16.5 ± 1.6^a^	0.01

Means within a column with different superscripts are different at:

A,B 0.05 *≤ P* < 0.01.

a-c *P* ≤ 0.05. All means are reported as means ± SEM, *n* = 6 at 12 birds (2 birds/pen × 6 pens).

### Skeletal Health

#### Bone Mineralization

**Figure 1:**
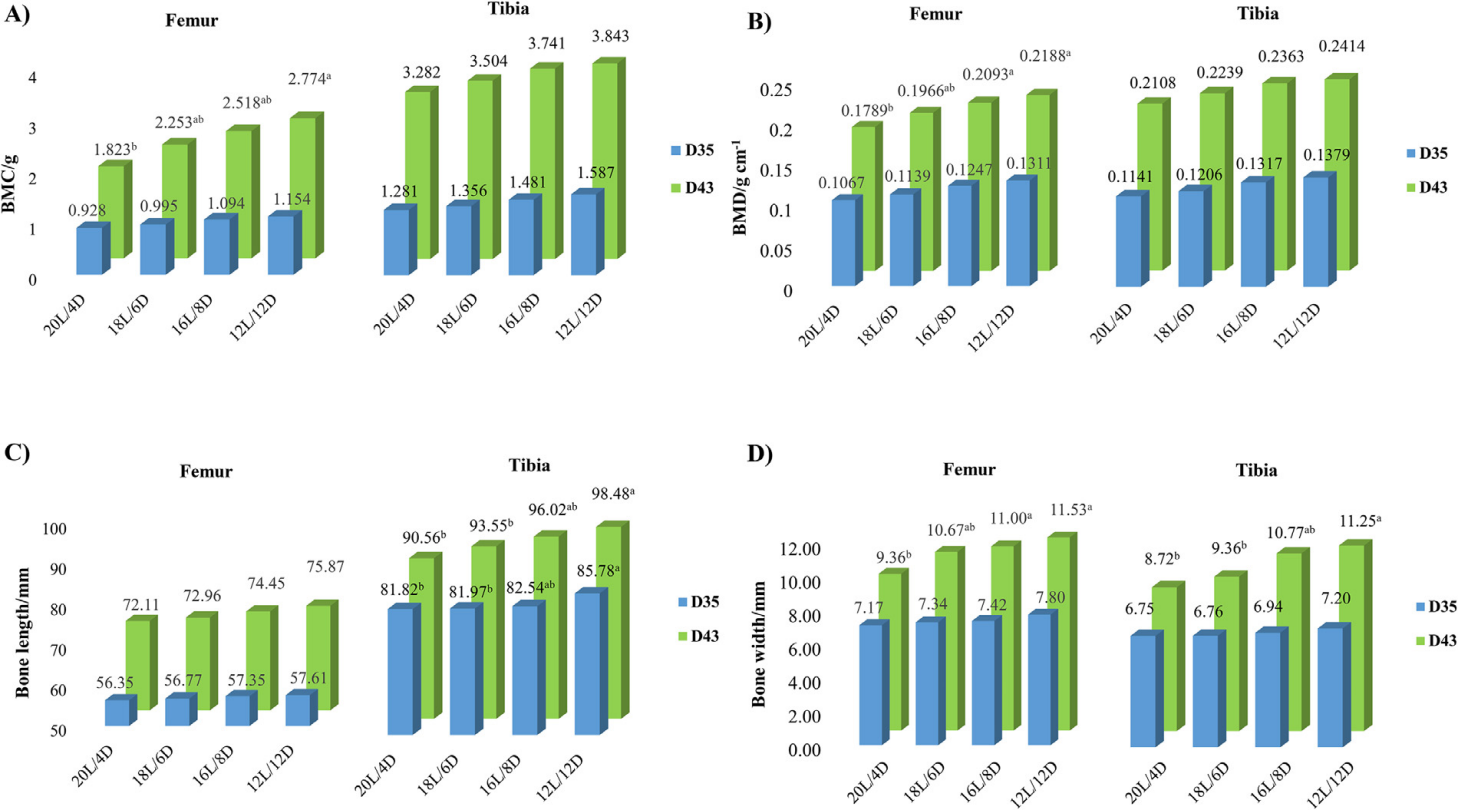
Effect of lighting program on bone health in broiler femur and tibia. (A) Bone mineral content (BMC), (B) Bone mineral density (BMD), (C) Bone length, and (D) Bone width. The means with different superscripts are different at: ^a,b^*P* < 0.05 (*n* = 6 at 12 birds, 2 birds/pen × 6 pens/treatment).

Compared to 20L birds, BMC was increased in both the femur and tibia of 12L birds at d 43 (*P* < 0.05) but not at d 35 (*P* > 0.05; Figure 1A). Also, BMC in the tibia of 12L birds was also higher than 18L birds (*P* < 0.05; Figure 1A). BMD was increased in the femur (*P* < 0.05) but not in the tibia of both 16L and 12L birds at d 43 compared to 20L birds (*P* > 0.05; Figure 1B). The tibia but not femur was much longer and wider in 12L birds compared to those of 18L and 20L birds at both d 35 and d 43 (*P* < 0.05, Figure 1C, D); while femoral width was larger in both 16L and 12L birds than that of 20L birds at d 43 only (*P* < 0.05, Figure 1D).

#### Gait Score

**Figure 2:**
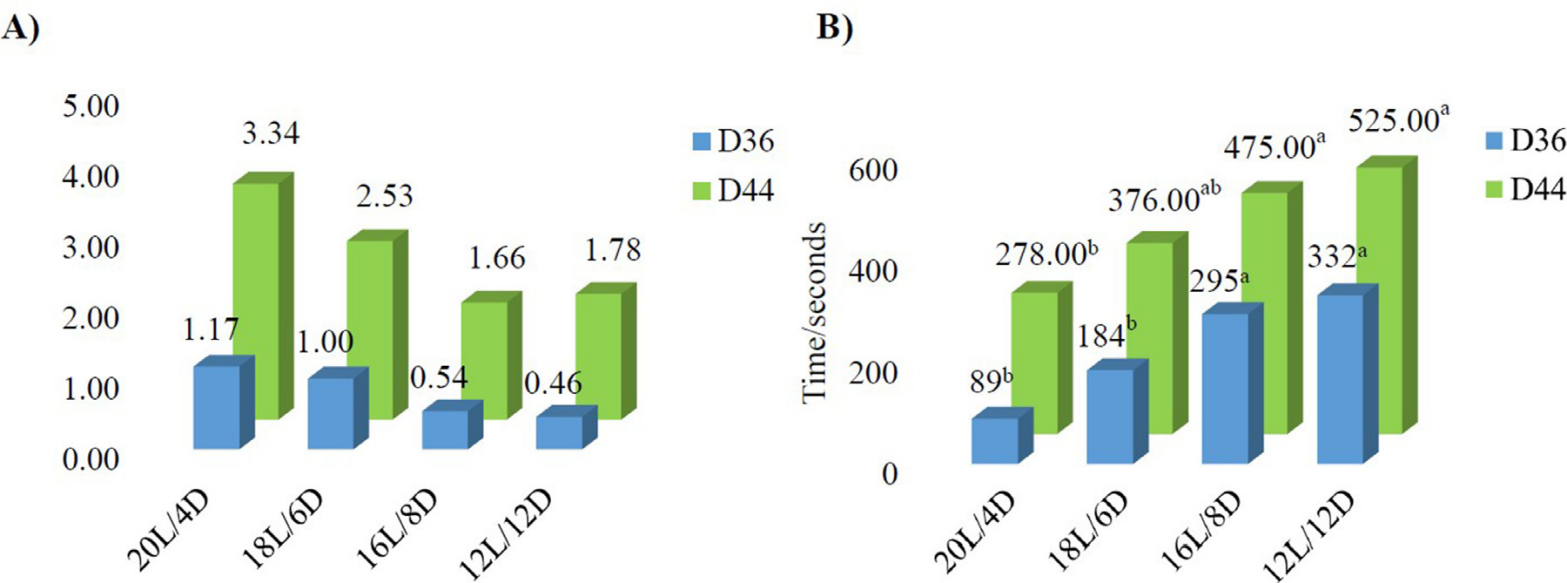
Effect of lighting program on bone strength in broilers. A) Gait score and B) latency to lie. The means with different superscripts are different at: ^a,b^*P* < 0.05 (*n* = 6 at 12 birds, 2 birds/pen × 6 pens/treatment).

There were no treatment effects on the GS in the broilers (*P* > 0.05, Figure 2A) at both d 36 and d 44 due to the most of broilers were categorized with a score of 0 (normal gait) and only a small proportion (<3%) identified with a score 1 or 2 (obvious and severe lameness) (the data were not shown).

#### Latency to Lie

12L and 16L birds stood much longer compared to both 18L and 20L birds at d 36 and compared to 20L birds at d 44 only (*P* < 0.05, Figure 2B).

### Stress and Fear Indicators

Both 20L and 18L birds had high H/L ratios than 16L and 12L birds at D 35 (21 d after the lighting treatment) (*P* < 0.05, Table 4), which was onward to d 43 (29 d after the light treatment) in 12L birds (*P* < 0.05) but not in 16L birds (*P* > 0.05). Furthermore, during the touch test, both 20L and 18L birds spent more latency time to reach a human presented in the pen compared to 16L and 12L birds at both d 35 (*P* = 0.07) and d 45 (*P* < 0.05). In addition, the time spent during the TI test was much longer in both 20L and 18L birds compared to 16L (*P* < 0.01) and 12L birds (*P* < 0.05) at d 45 (31 d after the lighting treatment).

**Table 4:** Effects of light programs on stress and fear indicators in broilers.

Treatment	H/L ratio		Touch test (%)		Tonic immobility (s)
	D 35	D 43	D 35	D 44	D 45
12L:12D	0.27^b^	0.31^b^	75.5^A^	71.3^b^	482^b^
16L:8D	0.31^b^	0.38^ab^	77.3^AB^	64.6^b^	323^c^
18L:6D	0.38^a^	0.42^a^	82.5^AB^	87.6^a^	727^a^
20L:4D	0.41^a^	0.46^a^	86.8^B^	93.3^a^	832^a^
SEM	0.02	0.03	4.20	7.67	56
*P* value	0.03	0.04	0.07	0.001	0.001

Means within a column with different superscripts are different at:

A,B 0.05 *≤ P* < 0.01.

a–c *P* ≤ 0.05. All means are reported as means + SEM created by mixed model analysis, *n* = 6 at 12 birds (2 birds/pen × 6 pens). An increased touch test score indicates a reduced fear of humans and an improved human-animal relationship.

## DISCUSSION

The lighting programs used in the intensive broiler production system have resulted in global animal health and welfare concerns (Kim et al., 2022; Shynkaruk et al., 2022). The present study examined the effects of various photoperiods on growth performance, skeletal health, stress response, and behavioral pattern in broilers. The data revealed that the lighting program at 12L as well as 16L improved broiler final growth with low stress levels compared to the rest photoperiod groups. Both 12L and 16L birds were also more active during the observation time with stronger femoral leg bones. Especially, 12L birds had heavier final BW and AWG, higher BMD and BMC with longer and wider femur, lower H/L ratios, and more birds reached to the observer during the touch test but spent a shorter time during the TI test.

In the current study, the used photoperiods have remarkable effects on the production performance of broilers in an age-specific manner. At d 20, 20L birds had a heavier BW with a tendency difference from 12L birds, while the difference was reversed with bird growth. 12L birds had heavier BW than 20L birds at d 35 and than both 20L and 18L birds at d 43. In addition, 12L birds as well as 16L birds had higher ADG from d 15 to d 43. The final BW was in the order: 3,304 g (12L) > 3,245 g (16L) > 3,206 g (18L) > 3,156 g (20L). There are several factors that could be involved in shaping growth patterns during the growth phases, including the biocircadian clock related physiological (the level of stress reaction and suppression of bone development) and behavioral changes revealed by the current study. Similarly, it has been reported that broiler health and welfare can be improved by reducing day length (Schwean-Landner et al., 2013). Schwean-Lardner and Classen (2010) also reported that compared to 23L, the optimized lighting, between 17L-20L, has positive effects on the growth rate, feed intake, and processing performance with good welfare indicators. Kim et al. (2022) also reported that 18L photoperiod, compared to 24L and 8L, improves broiler performance with lower stress levels and good welfare states. In another study, photoperiods of 2L, 4L, 6L, 8L, 12L, 15L, 18L, and 21L were used in broilers started at d 2 and reported that the photoperiods positively affected the feed intake and BW during the first 21 d, then became negatively when the photoperiods were beyond 12L up to 35 d of age (Lewis et al., 2009a). The greatest feed conversion efficiency was found in 12L birds. Similarly, it has been reported that longer dark periods (among 14L:10D, 17L:7D, 20L:4D, and 23L:1D) result in improved feed conversion (Shynkaruk et al., 2022). Classen et al. (2004) also indicated that longer darkness intervals (12L vs. 16L and 20L) improve bird feed efficiency with reduced metabolic diseases, feed intake, and growth rate at an early age but without affecting the final BW.

Several studies have shown that the changes in the external day-night cycle can disrupt the internal circadian clock, affecting behavioral patterns in humans and animals (Sanotra et al., 2002; Walker et al., 2020). In chickens, compared to a long day (24L), a short day (16L) significantly increases the proportion of eating, drinking, and locomotion (pecking, scratching, and dustbathing) in birds (Sanotra et al., 2002). Similarly, the current study reveals that mimicking natural lighting cycles, 12L as well as 16L:8D, has positive effects on behavioral exhibition in broilers. Both 12L and 16L birds spent more time locomoting, dustbathing, and standing but less sitting and sleeping during wk 3 to 4 compared to 18L and 20L birds; and these patterns were onward to wk 5 to 6 during the observation times, with increased eating and drinking in 12L birds. Similarly, it has been previously reported that darkness increases bird physical activity and walking ability. Sanotra et al. (2002) reported that reducing day length from 24L to 16L improves bird eating, drinking, and walking ability. Shynkaruk et al. (2019) also reported that feeding bout frequency was increased with the length of darkness. Increased exercise in 12L birds may be related to their stronger leg health as reported previously (Schwean-Lardner et al., 2012; Kaukonen, et al., 2017). Similarly, the heavier BW of 12L birds could be related to the birds spending more time to eat and lighting-related regulation in hormonal synthesis, gut development, and nutrient resorption (Schwean-Lardner et al., 2014; Yang et al., 2015; Shynkaruk et al., 2019).

In the current study, 12L and 16L broilers had higher BMD in the femur but not in the tibia at d 43. Also, BMC was higher in 12L birds compared to 20L birds. 12L birds also had greater leg bone development, that is, the femur length and femur and tibia width, compared to both 18L and 20L birds at d 43. The increased skeletal health could be related to 12L birds spending more time to eat as that feed is the major source of calcium in chickens. In addition, the higher bone quality could be related to the higher activeness found in 12L birds. Although there were no treatment effects on the gait score due to most of the birds at score 0, a normal level; 12L birds as well as 16L birds stood much longer during the LTL test compared to 20L birds. The LTL test has been routinely used in broilers to test their leg weakness, indicating the longer latency the stranger legs (Ruiz-Feria et al., 2014; Aydin, 2018). In one of our studies, it has reported that the 12L lighting program is beneficial in reducing muscle (*M. pectoralis* major) protein denaturation and improving lipid stability in birds compared to the birds under 20L, 18L, and 16L photoperiods (Tuell et al., 2020a,b). Similarly, Santotra et al. (2002) reported that a short lighting program (16L) increased bird walking ability with lower gait score and reduced the occurrence of tibial dyschondroplasia compared to a long lighting program (24L). Lewis et al. (2009b) also reported that tibial breaking strength peaked at 7L Ross broilers and 12L Cobb broilers among the broilers reared under 2L, 4L, 6L, 8L, 10L, 12L, 15L, 18L, or 21L photoperiod. Although the mechanisms underlying these changes are not investigated in this study, it could be related to previously reported increased osteogenesis during the light and suppressed bone resorption during the dark (Cui et al., 2021) as well as reduced stress-related osteolysis (Wei et al., 2022). In addition, these changes could be related to dysregulation of the 12-h light-dark cycle-associated perturbation of the gut microbial composition and their function in regulating neurohormone synthesis (Liang and FitzGerald, 2017; Deaver et al., 2018; Kim et al., 2019; Hong et al., 2020; Malik et al., 2020). Impaired sleep and loss of circadian rhythmicity affect the gut function and alter microbiota compassion via the microbiota-gut-brain axis (Reynolds et al., 2017; Wang et al., 2018a, 2022; Alvarez et al., 2020; Meyer et al., 2022). Several studies have revealed that maintaining optimal gut health is crucial for food digestion, calcium resorption, and nutrient metabolism by which it enhances skeletal health and welfare in animals (Schneeman, 2002; Skrypnik and Suliburska, 2018; Sharkey and Mawe, 2023).

Heterophil/lymphocyte ratio is a biological stress indicator, practically used to evaluate stress reactions (Gross and Siegel, 1983; Cheng et al., 2001; Lentfer et al., 2015; Zahorec, 2021) and related immunity (Thiam et al., 2021; Wang et al., 2023) in chickens. In this study, 12L and 16L birds had low H/L ratios compared to both 18L and 20L birds. The results may indicate the birds of 12L and 16L birds are less stressed. Similar results have been reported previously. Shynkaruk et al. (2022) reported that the shortest photoperiod (14L vs. 17L, 20L, and 23L) improves broiler welfare with reduced stress level indicated by the lowest H/L ratio. The reasons of this change are unknown but could be related to those reported in humans and other mammals. Periodic changes of the L-D cycle with extended light periods cause photoperiod stress, leading to increase the expressions of numerous oxidative stress response genes during the night (Abuelsoud et al., 2020; Wang et al., 2022). Naturally, light initiates a cascade of physiological events mediating the external inputs and interpretation of day length to the outputs of specific hormones, by which it determines whether the animals have prepared physically, physiologically, and behaviorally for the environmental changes (Bradshaw and Holzapfel, 2010; Gassen et al., 2019; Chen et al., 2020). A state of allostatic overload with the disrupted circadian clock may be developed, impairing the animals’ adaptability, disrupting the stress regulatory systems including the hypothalamic-pituitary-adrenal pathway and the sympathetic nervous system (Russell and Lightman, 2019; Mason et al., 2022). Profound changes in the autonomic nervous system followed prolonged exposure to short winter-like day lengths have been found in Siberian hamsters (Weil et al., 2009). These changes may affect the brain-gut-immune pathway in regulating immune cells (Elenko et al., 2000; He et al., 2003; Jakob et al., 2020). In addition, a recent study has reported that the change in H/L ratio is associated with the intestinal barrier function and immune response in infected chickens at d 7 and d 21 post *Salmonella* Enteritidis immune challenge, that is, birds with lower H/L ratios had enhanced immunity (Thiam et al., 2021, 2022). In addition, changes of the H/L ratios were correlated with the infection-caused alterations of cecal microbiota composition. Birds with low H/L ratios had a significantly higher abundance of Proteobacteria (*Escherichia coli*) and Bacteroidetes (*Bacteroides plebeius*) at d 7 and d 21 postinfection, respectively. The changes of Bacteroides were positively correlated with BW in the postinfected birds.

Several fear tests have been routinely used in poultry, such as TI and human touch tests as well as avoidance distance test, human approach test, novel arena, and novel object test (Graml et al., 2008; Rentsch et al., 2023). TI (animal hypnosis) is an all-or-none innate and reversible, natural defensive response; and TI test has become a common practice to measure the capability of an animal to avoid responses to shock, such as responding in a predator-prey confrontation in birds, by reducing neuronal activity (Campbell et al., 2019; Carli and Farabolini, 2022; Rentsch et al., 2023). The hippocampus is the center responding to stress-associated mental and emotional damage, and functionally involves TI onset and termination (Yousuf et al., 2020; Park et al., 2022). A state of intense fear is indicated by a longer duration of immobility. In this study, both 20L and 18L birds had longer immobility duration than 12L and 16L birds, which is evidence that changing 24-h L-D cycle can trigger fearfulness in chickens. Similar results have been reported in several studies. Sanotra et al. (2002) reported that birds reared under 16L had a shorter duration of TI compared to the birds housed under 24L. The conclusion that 12L birds are less stressed is supported by results from the human touch test which is another common fear-related test often used in captured animals including modern chickens (Vasdal et al., 2018; Bethell et al., 2019; Bertin et al., 2021). The outcome is the longer the time of withdrawal from the observer, the more fearfulness in the animals. In the current study, both 12L and 16L birds showed less latency time needed for becoming calmness and can be touched by the observers compared to 18L and 20L birds at both d 35 and d 44, especially at d 44. The results could be related to the birds adapting to the environment with less stress or birds becoming heavier and exhibiting fewer activities at the finish stage.

## CONCLUSIONS

The first part of this study investigated the photoperiod effects on broiler health and welfare. A short photoperiod, close to the natural light cycle, 12L:12D as well as 16L:8D broilers had improved production performance, leg bone health, and suppressed stress reaction compared to both 18L: 6D and 20L:4D broilers. Especially, 12L:12D birds had heavier final BW and AWG, higher BMD, BMC, longer and wider femur, with lower H/L ratios, and more birds reached the observer during the touch test and shorter latency time during the TI test. Taken together, our data suggest that providing 12 h of light per d may be more suitable for broiler production. Further studies will be conducted to investigate the cellular mechanisms underlying the photoperiod effects on broiler health and welfare.
